# Effects of CeO_2_ on the Si Precipitation Mechanism of SiCp/Al-Si Composite Prepared by Powder Metallurgy

**DOI:** 10.3390/ma13194365

**Published:** 2020-09-30

**Authors:** Bin Yang, Aiqin Wang, Kunding Liu, Chenlu Liu, Jingpei Xie, Guangxin Wang, Shizhong Wei

**Affiliations:** 1 Materials Science and Engineering School, Henan University of Science and Technology, Luoyang 471023, China; aiqin_wang@haust.edu.cn (A.W.); liukunding@haust.edu.cn (K.L.); liuchenlu@haust.edu.cn (C.L.); xiejp@haust.edu.cn (J.X.); wgx58@haust.edu.cn (G.W.); wsz@haust.edu.cn (S.W.); 2 Collaborative Innovation Center of Nonferrous Metals, Luoyang 471023, China

**Keywords:** powder metallurgy, rare earth element, precipitated Si, microstructure refinement

## Abstract

SiCp/Al-Si composites with different CeO_2_ contents were prepared by a powder metallurgy method. The effect of CeO_2_ content on the microstructure of the composites was studied. The mechanism of CeO_2_ on the precipitation of Si during sintering was analyzed by theoretical calculations. The results show that the appropriate amount of CeO_2_ can significantly refine the size of precipitated Si particles in the composite and increase the number of Si particles. With the increase of CeO_2_ content from 0 to 0.6 wt%, the number of Si particles precipitated in the composites increases gradually, and the average particle size of Si particles decreases gradually. When the CeO_2_ content is 0.6 wt%, the number of Si particles precipitated in the composites reaches the maximum, and the average particle size reaches the minimum. However, with the increase of CeO_2_ content from 0.6 wt% to 1.8 wt%, the number of Si particles precipitated in the composites began to decrease, and the average size of Si particles gradually increased. CeO_2_ can be used as heterogeneous nucleation substrate of precipitated Si, and the nucleation rate of precipitated Si on a CeO_2_ substrate is higher than that on an aluminum substrate. The proper addition of CeO_2_ can improve the nucleation efficiency of precipitated Si, thus increasing the amount and refining the size of precipitated Si.

## 1. Introduction

With the development of the modern manufacturing industry, research and development of high-performance materials has become one of the important topics in the development of the aerospace industry [1,2,3]. In spacecraft design, due to the particularities of the space flight environment, the materials used for the vessel and its auxiliary equipment are usually required to have ultra-high specific strength and toughness, good high-temperature performance and thermal stability, as well as remarkable fatigue resistance, vibration resistance, corrosion resistance, etc. [4,5,6,7].

Due to the advantages of low density, high specific strength, low thermal expansion coefficient, good thermal conductivity and high wear resistance, SiC particle reinforced Al-Si matrix composite (SiCp/Al-Si composite) has been widely used in aerospace applications, electronic instruments, military equipment, wear-resistant materials and many other fields [8,9,10,11,12]. SiCp/Al-Si composites prepared by powder metallurgy usually have the following advantages: the distribution of the reinforcement phase is uniform, the content is easy to adjust, the proportion of each component is accurate and there is no obvious agglomeration [13,14]. In addition, due to the low temperature used in the preparation process, the composites are generally in solid or partial melting state, so the interfacial reaction between matrix and reinforcement is very weak, which reduces the formation of interface impurities and brittle phases [15]. However, for the SiCp/Al-Si composites with high Si content in the matrix, multi-scale precipitation of Si phase will occur during the preparation process, and coarse and irregular Si particles will be precipitated in the matrix [16,17,18]. These particles can easily split the matrix, reduce the mechanical properties of the materials and restrict the improvement of its physical and mechanical properties. Therefore, it is of great significance to improve the physical and mechanical properties of SiCp/Al-Si composites by reducing or preventing the multi-scale precipitation of a Si phase during the preparation process.

At present, many scholars have studied the application of rare earths as grain refiner in casting Al-Si alloys [19,20,21,22]. Compared with other rare earth elements, Ce has been the most investigated due to its relatively low cost and good compatibility with the aluminum matrix. Li et al. [23] found that the addition of 1.0 wt% Ce significantly refined the primary Si and transferred the morphology from coarse irregular to fine blocky. Xue et al. [24] reported that CeO_2_ additive could significantly improve the dispersion of in-situ formed TiB_2_ in an Al matrix while refining α-Al grains. Wu et al. [25] fabricated Ti/Al_2_O_3_ composites with different volume content of CeO_2_ via vacuum hot-pressing sintering and found that the addition of CeO_2_ could significantly improve the microhardness, flexural strength and fracture toughness. However, there are few reports on the application of CeO_2_ in Al-Si alloy and Al-Si alloy matrix composites prepared by powder metallurgy and little attention has been paid to the effects of CeO_2_ on the microstructure of SiCp/Al-Si composites. In this paper, SiCp/Al-Si composites with different CeO_2_ contents were prepared by a powder metallurgy method, the influence of CeO_2_ content on the microstructure of the composites were studied and the mechanism of the influence of CeO_2_ on the precipitation of Si phase in the composites was discussed.

## 2. Experimental Process

The SiCp/Al-Si composites with different CeO_2_ contents were prepared by a powder metallurgy method. Al-19Si-1.5Cu-0.6Mg alloy powder prepared by the gas-atomization method was used as matrix, SiCp with average particle size of 10 μm was selected as reinforcement material and its mass fraction was 20 wt%, The additive used in the experiments was high purity CeO_2_ powder, which mass fraction was respectively 0, 0.15 wt%, 0.3 wt%, 0.6 wt%, 1.2 wt%, 1.8 wt%. First, the above powders were mixed by a ball mill (the ratio of balls to powder was 2:1) for 4 h and then dried. Next the mixed powders were cold-rolled to a diameter of 78 mm, length 48 mm billet with 500 MPa pressure in a hydraulic machine; then the billets were heated in a tube furnace, the sintering temperature was 550 ℃, sintering time was 3 h (the protective gas was N_2_).

The X-ray diffraction (XRD) patterns of composites with different CeO_2_ contents were determined using an X-pertpro X-ray diffractometer (PANalytical, Eindhoven, The Netherlands) with Cu K_α_ radiation (k = 0.15406 nm) operated at 40 kV and 100 mA. The microstructure of the samples were observed using scanning electron microscopey (SEM, JSM-5600LV, JEOL, Tokyo, Japan) with energy dispersive spectroscopy (EDS, Kevex, Texas, TX, USA) and transmission electron microscopy (TEM, JEOL JEM-2100). Samples for SEM analysis were prepared via grinding with SiC abrasive papers and polishing with an Al_2_O_3_ suspension solution and diamond solutions of different abrasive sizes (6, 3, 1 and 0.3 μm). After metallographic polishing, the samples were corroded by Keller reagent for 30 s. The TEM foil was mechanically polished to about 20 μm and further thinned via ion milling with a precision ion polishing system (PIPS, Model 691, Gatan, Pleasanton, CA, USA). The average grain size of Si particles precipitated from the composites with different CeO_2_ content was measured by Image Pro Plus software v6.0. This software generates histograms of precipitated Si particles from the SEM image and quantifies the diameter of precipitated Si through line profile analysis which provides the average grain size of all precipitated Si particles.

## 3. Results and Discussion

### 3.1. The XRD Analysis of SiCp/Al-Si Composites with Different CeO_2_ Contents

**Figure 1:**
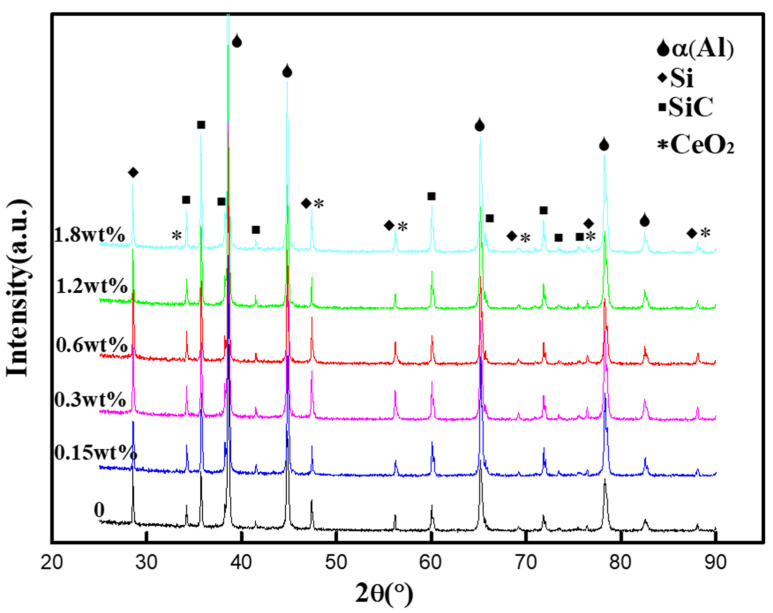
XRD pattern of SiCp/Al-Si Composites with different CeO_2_ Contents.

Figure 1 shows the XRD patterns of SiCp/Al-Si Composites with different CeO_2_ contents. It can be seen that all the samples contain the diffraction peaks of Al matrix (JCPDS card No.04–0787), SiC particles (JCPDS card No. 29–1131) and precipitated Si (JCPDS card No. 27–1402). However, due to the relatively low amount of addition and the fact the diffraction peaks of CeO_2_ overlap with those of precipitated Si, the diffraction peak of CeO_2_ (JCPDS card No. 34–0394) is only found in the composites with 1.8 wt% CeO_2_ content, and the diffraction peak intensity is weak. This shows that CeO_2_ added in the composite is relatively stable, and CeO_2_ does not react during the preparation process.

### 3.2. Effect of CeO_2_ Additions on the Microstructure of SiCp/Al-Si Composites

**Figure 2:**
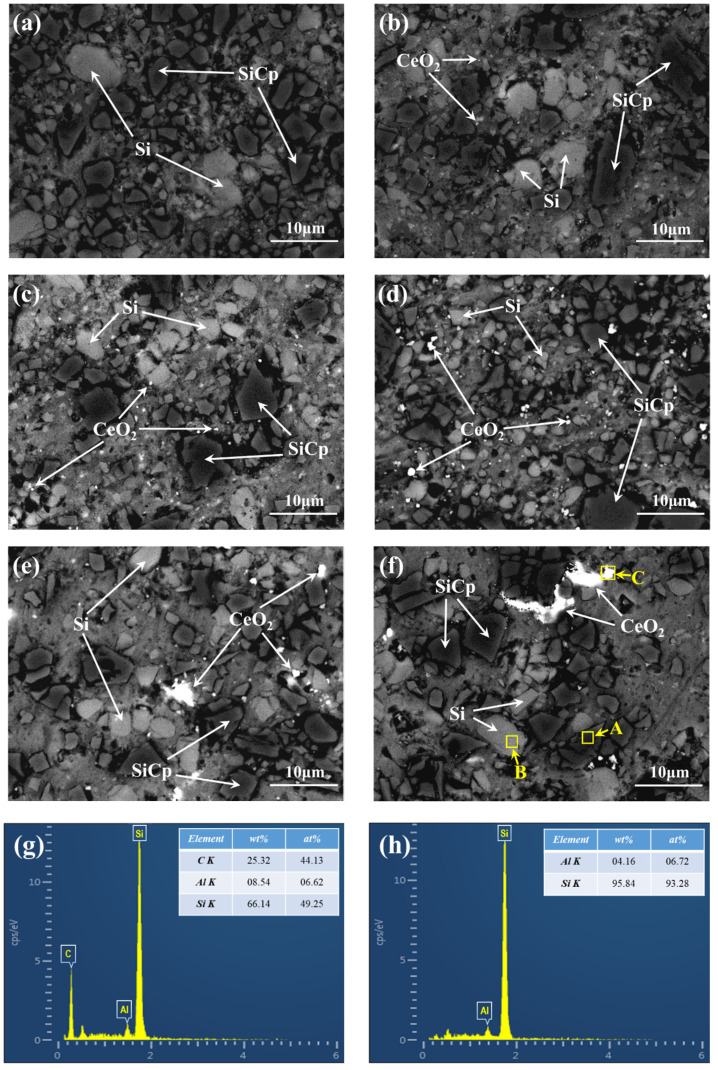

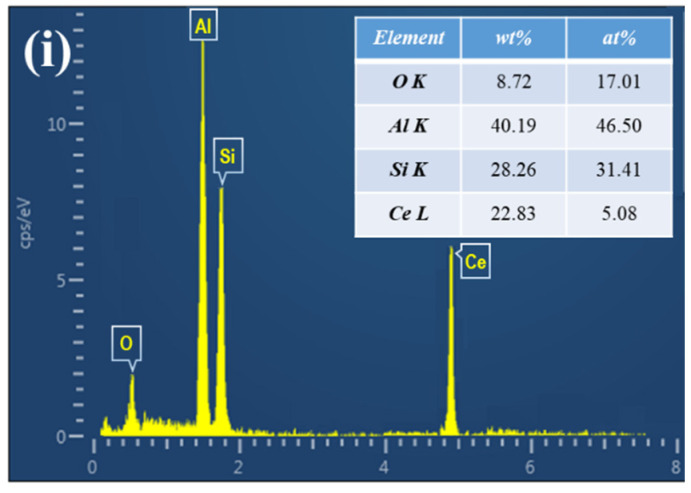
The SEM and EDS pictures of SiCp/Al-Si Composites with different CeO_2_ Contents: (**a**) 0; (**b**) 0.15 wt%; (**c**) 0.30 wt%; (**d**) 0.60 wt%; (**e**) 1.20 wt%; (**f**) 1.80 wt%; (**g**) the energy spectrum diagram of region A in Figure 2f; (**h**) the energy spectrum diagram of region B in Figure 2f; (**i**) the energy spectrum diagram of region C in Figure 2f.

Figure 2 shows the SEM and EDS images of SiCp/Al-Si Composites with different CeO_2_ contents. It can be seen that the microstructure of the composites mainly consists of three phases: a dark gray particle phase (as shown in region A in Figure 2f), a light gray particle phase (as shown in region B in Figure 2f) and a white particle phase (as shown in region C in Figure 2f). Combining the XRD analysis in Figure 1 and the EDS results in Figure 2f–i, it can be concluded that the dark gray particle phase in the composites is SiCp, the light gray particle phase is precipitated Si and the white particle phase is CeO_2_. 

**Figure 3:**
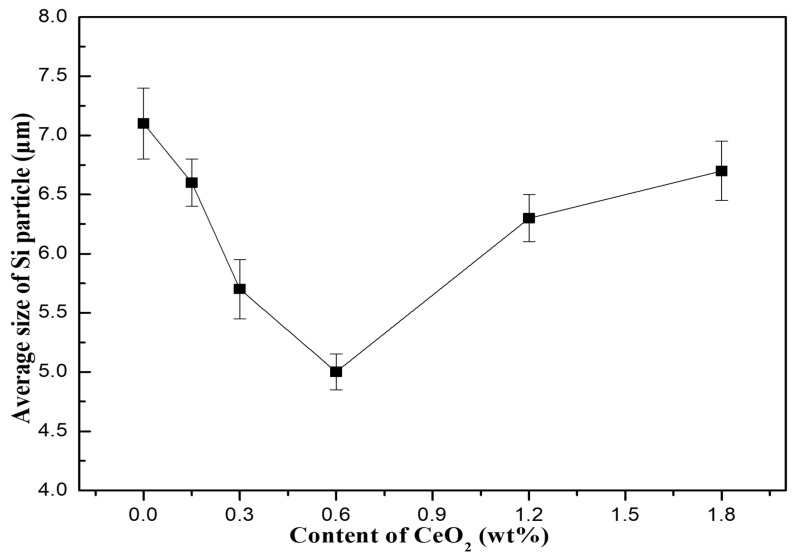
Average size of Si particles precipitated from SiCp/Al-Si composites with different contents of CeO_2_.

The average diameter of Si particles precipitated in the composites with different CeO_2_ contents is shown in Figure 3. It can be seen that when the content of CeO_2_ is between 0 and 0.6 wt%, the average size of precipitated Si particles gradually decreases with the increase of CeO_2_ content, and the number of precipitated Si particles gradually increases; when the content of CeO_2_ is between 0.6 wt% and 1.8 wt%, the change trend of average size and number of precipitated Si particles is opposite to the above, the main reason for this phenomenon may be that the agglomeration of CeO_2_ increases with the increase of CeO_2_ content. When the content of CeO_2_ is less than 0.6 wt%, CeO_2_ mainly exists in the form of particles, and when the content of CeO_2_ is more than 0.6 wt%, the agglomeration of CeO_2_ in the composite increases. Obviously, an appropriate amount of CeO_2_ can refine the precipitated Si, When the CeO_2_ content is 0.6 wt%, the number of Si particles precipitated in the composites reaches the maximum and the average particle size reaches the minimum. 

The existence of CeO_2_ can slow down the diffusion of precipitated Si atoms in Al matrix, thus reducing the growth rate of precipitated Si and refining the particles. The calculated results about the average diameter of Si precipitated in composites are consistent with the microstructure analysis. The morphological difference of the precipitated Si particles was relatively small, which indicates that CeO_2_ content has little effect on the morphology of precipitated Si particles.

### 3.3. Influence Mechanism of CeO_2_ on the Microstructure of SiCp/Al-Si Composites

**Figure 4:**
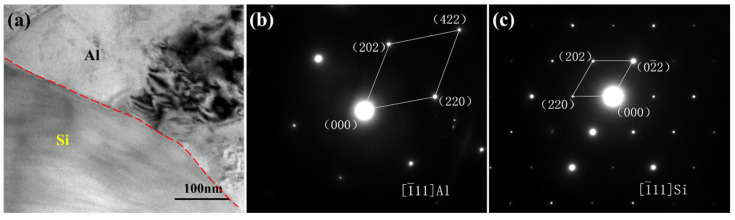
TEM observation of SiCp/Al-Si composites without CeO_2_: (**a**) Al-Si interface; (**b**) Diffraction patterns of the Al matrix; (**c**) Diffraction patterns of the precipitated Si.

**Figure 5:**
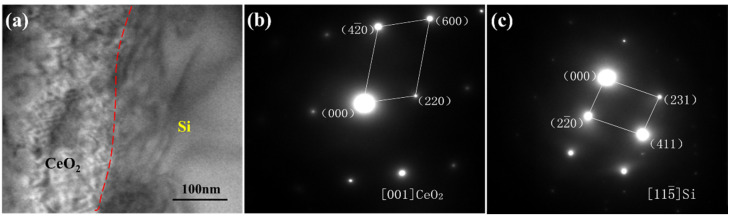
TEM observation of SiCp/Al-Si composites with 0.6 wt% CeO_2_ content: (**a**) CeO_2_-Si interface; (**b**) Diffraction patterns of the CeO_2_; (**c**) Diffraction patterns of the precipitated Si.

In order to study the influence mechanism of CeO_2_ on the size and quantity of precipitated Si in the composites, the composite without CeO_2_ and the composite with 0.6 wt% CeO_2_ content were analyzed by TEM. Figure 4 shows the TEM microstructure and corresponding electron diffraction patterns of the SiCp/Al-Si composite without CeO_2_. After calibration by the software MDI jade 5.0, the electron diffraction patterns should be Al and precipitated Si, respectively. This indicates that the Al matrix is the main nucleation substrate for the precipitated Si in the composites without CeO_2_. Figure 5 shows the TEM microstructure and corresponding electron diffraction patterns of the SiCp/Al-Si composite 0.6 wt% CeO_2_ content, and diffraction patterns calibration indicated that they should be CeO_2_ and precipitated Si, respectively. It can be concluded that in addition to Al matrix as the nucleation substrate, CeO_2_ can also be used as the nucleation substrate for precipitated Si in the composites containing CeO_2_.

According to the analysis of TEM images and the corresponding electron diffraction patterns of the composites, it can be determined that CeO_2_ could be used as the nucleation substrate of precipitated Si, so as to improve the nucleation rate and refine the size of precipitated Si particles. Table 1 shows the basic physical parameters of precipitated Si, CeO_2_ and Al. It can be seen that the precipitated Si, CeO_2_ and Al matrix belong to the face-centered cubic crystal system. However, the difference of lattice constant values between precipitated Si and Al is larger than that of precipitated Si and CeO_2_.

Turnbull and Vonnegut first proposed that the heterogeneous nucleation efficiency of nucleation substrate depends on the lattice mismatch between the nucleation substrate and the nucleation phase [26]. The theory holds that the smaller the mismatch degree between nucleation substrate and nucleation phase is, the more lattice matching between nucleation substrate and nucleation phase is, and the energy caused by lattice mismatch between nucleation substrate and nucleation phase is smaller, too. That is, the smaller the interface energy between nucleation substrate and nucleation phase is, the higher nucleation rate of nucleation phase is. Because the precipitated Si, CeO_2_ and Al are face centered cubic structure, the mismatch degree of precipitated Si and two nucleation substrates can be expressed by one-dimensional mismatch degree:

(1) $$\delta =\left|a_{s}-a_{n}\right|/a_{n}$$

In Equation (1), *a_s_* is the lattice constant of nucleation substrate, Å; and *a_n_* is the lattice constant of nucleation phase, Å. Substituting the values in Table 1 into Equation (1), the calculation shows that the mismatch degree of precipitated Si and CeO_2_ is 0.22, and that of precipitated Si and Al is 0.64. According to the lattice mismatch theory, it can be concluded that the nucleation rate of the precipitated Si with CeO_2_ as the nucleation substrate is greater than that with Al as the nucleation substrate because the mismatch degree of the precipitated Si and CeO_2_ is less than that of the precipitated Si and Al. In addition, according to the crystal growth theory of solid phase transformation [27], the growth mechanism of precipitated Si is mainly diffusion-controlled growth. Although the content of CeO_2_ in the composites is relatively low, the existence of CeO_2_ can still slow down the diffusion of precipitated Si atoms in Al matrix, thus reducing the growth rate of precipitated Si and refining the particles.

### 3.4. Calculation of Nucleation Rate of Precipitated Si in Composites

The Al-Si alloy powder used in this study is supersaturated solid solution prepared by rapid solidification method, the precipitation process of solid solution Si during sintering is a solid phase transformation process, and the nucleation process of precipitated Si in composite materials belongs to heterogeneous nucleation. Compared with homogeneous nucleation, heterogeneous nucleation requires less energy, the formula of free energy difference in heterogeneous nucleation system is as follows:

(2) $$\Delta G_{i}=V\Delta G_{V}+A\gamma_{i}+V\Delta G_{si}-\Delta G_{di}$$

In Equation (2), Δ*G_i_* is the system free energy, J; Δ*G_V_* is the unit volume free energy of precipitated Si, J/cm^3^; *V* is the volume of precipitated Si particles, cm^3^; *γ_i_* is the unit area interface energy between the precipitated Si and the nucleation substrate, J/cm^2^; *A* is the interface area, cm^2^; Δ*G_si_* is the unit volume elastic strain energy, J/cm^3^; and Δ*G_di_* is the surface energy at defects, J.

It is generally believed that *V*Δ*G_V_* is nucleation driving force, *Aγ_i_ + V*Δ*G_si_* is resistance to nucleation, and Δ*G_di_* is the surface energy at non-equilibrium defects, which contributes to nucleation work and promotes nucleation. For the convenience of calculation, it is assumed that the interface area between nucleation and nucleation substrate is equal to the surface area of nucleation, therefore, Δ*G_di_* can be expressed as:

(3) $$\Delta G_{di}=A\sigma_{i}$$

where *σ_i_* is the unit area surface energy of different nucleation substrates, J/cm^2^. Hence, Equation (2) can be rewritten as:

(4) $$\Delta G_{i}=V\Delta G_{V}+A\gamma_{i}+V\Delta G_{si}-A\sigma_{i}$$

Therefore, the critical nucleation size *r_ki_* and the critical nucleation Energy Δ*G_ki_* of precipitated Si could be identified as Equations (5) and (6):

(5) $$r_{ki}=-2\left(\gamma_{i}-\sigma_{i}\right)/\left(\Delta G_{V}-\Delta G_{si}\right)$$

(6) $$\Delta G_{ki}=-16\pi {\left(\gamma_{i}-\sigma_{i}\right)}^{3}/3{\left(\Delta G_{V}-\Delta G_{si}\right)}^{2}$$

The nucleation rate for heterogeneous nucleation could be identified as Equation (7):

(7) $$N_{i}=N_{0}exp\frac{-\Delta G_{ki}}{kT}exp\frac{-\Delta G_{A}}{kT}$$

In Equation (7), *N_i_* is the nucleation rate of different nucleation substrates, 1/(s·cm^3^); *k* is Boltzmann’s constant, *T* is absolute temperature, K; Δ*G_A_* is the diffusion activation energy, J; and *N*_0_ is the atomic number of precipitated Si per unit volume, 1/(s·cm^3^). Substituting Equation (6) into Equation (7), then the Equation (8) can be rewritten as:

(8) $$N_{i}=\frac{N_{0}kT}{h}exp\frac{-\frac{16\pi {\left(\gamma_{i}-\sigma_{i}\right)}^{3}}{3{\left(\Delta G_{V}-\Delta G_{si}\right)}^{2}}}{kT}exp\frac{-\Delta G_{A}}{kT}$$

where *h* is Planck constant.

Table 2 shows the parameter values for the nucleation of precipitated Si. According to the data in Table 2, it can be calculated that *N_CeO2_ =* 4.66 × 10^12^(s·cm^3^)^−1^, *N_Al_ =* 3.33 × 10^12^(s·cm^3^)^−1^, *N_CeO2_* > *N_Al_*. Therefore, it can be concluded by calculation that the nucleation rate of precipitated Si on CeO_2_ substrate is higher than that on Al substrate. However, it should be noted that the nucleation rate of precipitated Si on CeO_2_ substrate is not much higher than that on Al substrate. Because of the value of areal surface energy *σ_i_* for Al matrix (1 × 10^−4^) is higher than that of CeO_2_ (1 × 10^−5^), it can be concluded by Equation (5) that the critical radius of Si with CeO_2_ as nucleation substrate is smaller than that with Al matrix as nucleation substrate. The decrease of the critical nucleation radius of precipitated Si will lead to the decrease of critical nucleation energy and the increase of nucleation rate. This is consistent with our calculation. The addition of CeO_2_ can not only provide more nucleation substrates for precipitated Si to improve the nucleation rate, so as to refine the size and increase the number of precipitated Si particles. Combined with the previous analysis, it can be concluded that when the content of CeO_2_ is less than 0.6 wt%, the refining effect is gradually enhanced with the increase of CeO_2_ content, which happens because there are more and more CeO_2_ as the nucleation substrate of precipitated Si, and the existence of CeO_2_ can slow down the diffusion of precipitated Si atoms in Al matrix, thus reducing the growth rate of precipitated Si and refining the particles. However, When the content of CeO_2_ is higher than 0.6 wt%, the refining effect is gradually weakened, which is due to the relatively serious agglomeration phenomenon caused by excessive CeO_2_ content, weakens the ability of hindering the growth of precipitated Si, and reduces the refining effect.

## 4. Conclusions

(1) SiCp/Al-Si composites with different CeO_2_ contents were successfully prepared by a powder metallurgy method. When the content of CeO_2_ is less than 0.6 wt%, CeO_2_ mainly exists in the form of discrete particles and when the content of CeO_2_ is more than 0.6 wt%, the agglomeration of CeO_2_ increases.
(2) An appropriate amount of CeO_2_ can obviously refine the size of precipitated Si particles and increase the amount of Si particles. With the increase of CeO_2_ content from 0 to 1.8 wt%, the number of precipitated Si particles first increases and then decreases, and the average size of precipitated Si particles first increases and then decreases, too. When the CeO_2_ content is 0.6 wt%, the number of Si particles precipitated in the composites is the largest and the average size is the smallest.
(3) For the composite without CeO_2_, the nucleation of precipitated Si is mainly based on Al matrix. The addition of CeO_2_ can be used as the heterogeneous nucleation substrate for precipitated Si, which improves the nucleation rate of precipitated Si. Moreover, the nucleation rate of precipitated Si on CeO_2_ substrate is higher than that on Al substrate, which further improves the nucleation rate of precipitated Si, thus increasing the number of precipitated Si particles and refining the size of precipitated Si particles.

## Figures and Tables

**Table 1:** Basic physical parameter of Si, CeO_2_ and Al.

Phase	Melting Point (K)	Crystal Structure	Lattice Constant (Å)
Si	1673	FCC	0.6636
CeO_2_	2873	FCC	0.5411
Al	933	FCC	0.4050

**Table 2:** The parameter value for the nucleation of precipitated Si on different nucleation substrates.

Parameter Value		Nucleation Substrates	
		Al Matrix	CeO_2_
ΔGv	(J/cm^3^)	10^4^	10^4^
ΔG_A_	(J)	2.19 × 10^−19^	2.19 × 10^−19^
ΔG_si_	(J/cm^3^)	0	15
N_0_	((s·cm^3^)^−^^1^)	3.94 × 10^21^	3.94 × 10^21^
γ_i_	(J/cm^2^)	4 × 10^−6^	6 × 10^−6^
σ_i_	(J/cm^2^)	1 × 10^−4^	5 × 10^−5^
k	(J/k)	1.38 × 10^−23^	1.38 × 10^−23^
h	(J·s)	6.62 × 10^−34^	6.62 × 10^−34^
T	(K)	823	823
